# A case of torsed wandering spleen presenting as perforated acute appendicitis: A case report and literature review

**DOI:** 10.1016/j.ijscr.2025.111421

**Published:** 2025-05-09

**Authors:** Ayat Aljuba, Alaa R. AL-Ihribat, Bahaa AbuRahmeh, Noor Khashan, Kareem lbraheem, Yara al Najajreh

**Affiliations:** a Faculty of Medicine, Palestine Polytechnic University, Hebron 90200, Palestine; b Palestinian Clinical Research Center, Bethlehem, Palestine; c Faculty of Medicine, Al-Quds University, Jerusalem, Palestine

**Keywords:** Wandering spleen, Splenic torsion, Acute abdomen, Pediatric splenectomy, Right lower quadrant pain

## Abstract

**Introduction:**

Wandering spleen (WS) is a rare circumstance characterized via exaggerated splenic mobility because of absent or lax suspensory ligaments. It predisposes the spleen to torsion, that could purpose infarction, rupture, or gangrene, requiring urgent intervention.

**Presentation of case:**

We report a 10-year-old girl with a three-day history of acute abdominal pain, vomiting, and fever. Physical examination revealed diffuse tenderness, predominantly in the right lower quadrant (RLQ), mimicking appendicitis. Ultrasound and CT showed WS with torsion, showing the characteristic whirl sign. Emergency laparotomy revealed a 720-diploma splenic torsion with necrosis, necessitating splenectomy. The patient had an uneventful recovery and was discharged on postoperative day five.

**Discussion:**

WS is an extraordinary entity (<0.2 % occurrence) with a bimodal age distribution, often affecting adolescents and girls. It can be congenital or acquired and presents variably, from asymptomatic cases to acute abdomen. Torsion leads to vascular compromise, requiring prompt surgical intervention. This case was very interesting to the surgical team for its atypical presentation and its resemblance to perforated appendicitis.

**Conclusion:**

WS must be considered in cases of acute abdomen with an absent spleen on imaging. Early recognition and surgical intervention are essential to prevent complications in patients with acute abdomen. Ultrasound and CT are critical for early detection and differentiation from different abdomen emergencies.

## Highlights

• Mimicked Appendicitis: The patient presented with right lower quadrant pain, fever, and peritoneal signs, initially suggesting ruptured appendicitis rather than splenic torsion.
• Rare in Pediatric Females: Wandering spleen is uncommon in children and typically affects males, making its occurrence in a 10-year-old girl unusual.
• Severe 720° Torsion with Necrosis: The spleen twisted completely on its pedicle, leading to vascular compromise, necrosis, and the need for emergency splenectomy.
• Crucial Role of Imaging: Ultrasound revealed an absent spleen in its normal position, and CT confirmed torsion with a “whirl sign,” emphasizing imaging’s importance in differentiating acute abdominal conditions.

## 1 Introduction

Wandering spleen (WS), also known as ectopic spleen, is a rare entity that results from abnormal mobility of the spleen due to absence or laxity of its suspensory ligaments, which leads to abnormal location of the spleen away from the typical left hypochondriac region [1]. The incidence of WS is estimated to be <0.2 % of the population, with a higher prevalence observed in children and women of reproductive age [1,2]. With a different clinical presentations, a 30–50 % of patients presenting with sudden splenic torsion [3], which may results in acute abdomen with serious complications such as splenic infarction, rupture, gangrene, or abscess with a 50 % mortality rate [4]. Once diagnosis confirmed by imaging, usually abdominal ultrasound or CT, surgical intervention is required either by splenopexy or splenectomy depending on the viability of the organ and can be done laparoscopically or by laparotomy [5]. We present a 10-year-old girl with acute generalized abdominal pain, imaging identified an ectopic spleen with splenic torsion, confirmed by CT scan, which is characterized by the whirl sign.

Emergency laparotomy revealed a 720-degree splenic torsion with necrosis, necessitating splenectomy. The patient showed uneventful recovery and discharged with a good general condition.

## 2 Case presentation

This case report adheres to the SCARE 2023 guidelines for surgical case reporting [6].

A 10 year-old girl presented to the emergency department with a 3-day history of generalized acute abdominal pain associated with non-bilious vomiting and fever 38 °C (recorded by the family). She has had a history of recurrent brief attacks dull abdominal pain in the RLQ with sudden onset and offset. She did not have any history of trauma, with unremarkable medical and surgical history.

On physical examination, the abdomen was rigid with diffuse tenderness and rebound tenderness, which was mainly found on the right lower quadrant. Otherwise unremarkable cardiorespiratory examination. Her body temperature was 37.2 °C, heart rate was 130 beats/min, respiratory rate was 24 breaths/min, and blood pressure was 112/87 mmHg. Laboratory tests showed that hemoglobin was 10.6 g/dL, white blood cells count was 11.3 × 10^9^/L and CRP was 92 mg/L. The other laboratory tests were within normal range.

**Fig. 1:**
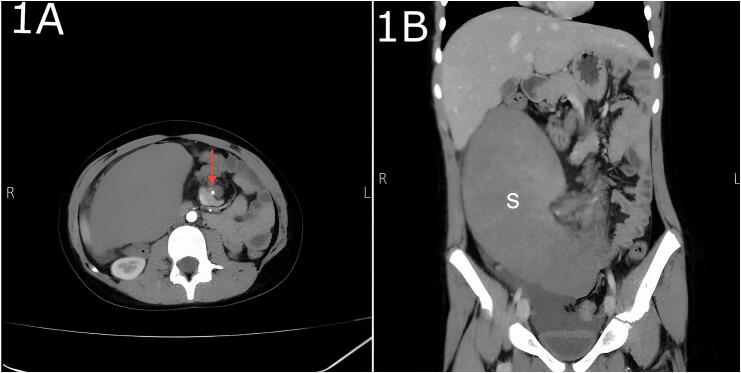
A: Axial contrast-enhanced CT image demonstrates a whorled appearance of the splenic vessels (red arrow), indicative of vascular torsion in a case of wandering spleen. This suggests splenic volvulus, a potential surgical emergency due to the risk of infarction. B: This contrast-enhanced coronal CT scan shows an ectopic spleen displaced into the right lower abdomen/pelvis, indicative of wandering spleen due to the absence or laxity of normal splenic ligamentous attachments. (For interpretation of the references to color in this figure legend, the reader is referred to the web version of this article.)

Abdominal ultrasound showed absence of spleen at its usual site, however a hyperechoic mass with a diameter 19 cm identified in the right lower abdominal region which was suggestive ectopic spleen and the Doppler showed absence of color flow. Contrast-enhanced CT scan of the abdomen was performed and revealed that the spleen was located in the right lower abdomen with swirling of the twisted splenic pedicle, which is shown as the whirl sign, and the splenic torsion was confirmed (Fig. 1).

**Fig. 2:**
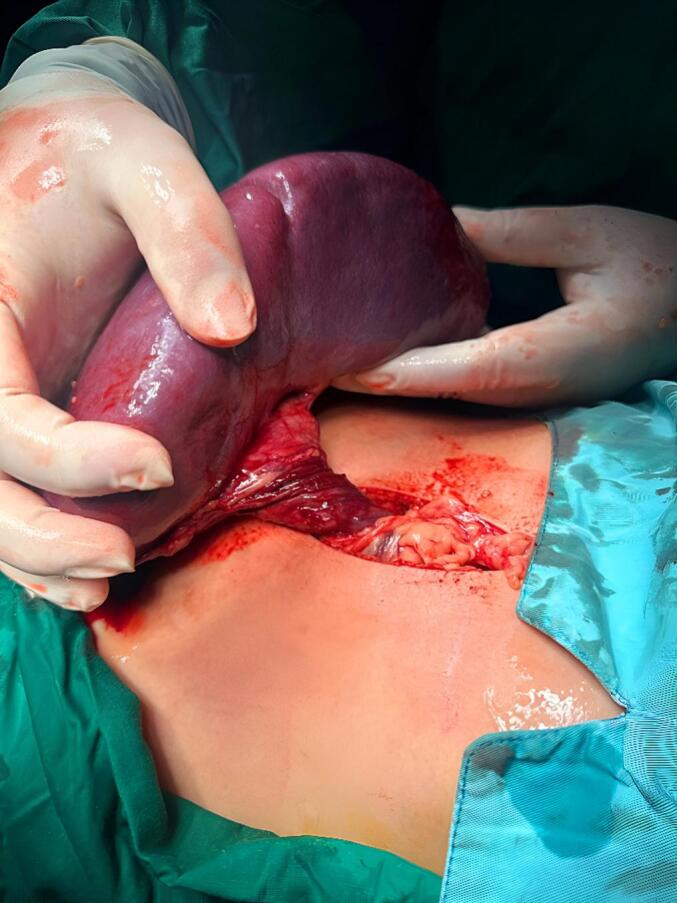
Intraoperative appearance of twisted splenic 720° counterclockwise around its pedicle and infarcted spleen.

Splenic torsion was confirmed and the patient was prepared for emergency laparotomy. During the exploratory surgery, the absence of ligamentous attachments of the spleen was confirmed and the spleen was torsed 720° on its vascular pedicle (Fig. 2). At a first glance, the spleen appeared dusky, had lost its capsular sheen, and was severely enlarged, suggesting a splenic infarction (Fig. 2). To give a chance to the spleen, a complete detorsion of the vascular pedicle with reperfusion of blood was done for about 1 h; however, the spleen remained necrotic, showing no signs of perfusion; therefore a splenectomy was performed in a forced manner. The operation was successful, without any complication. Postoperatively, the patient looked well, and had an uneventful recovery course, and the patient was discharged on the 5th day postoperatively.

## 3 Discussion

Wandering spleen is a condition characterized by greater laxity or complete absence of the splenic ligaments, which makes the spleen hypermobile and can migrate to any area of the abdomen or pelvis [7]. Normally, the spleen is located in the left hypochondrium attached to the stomach and posterior abdominal wall via gastrosplenic and the splenorenal ligaments, and it is supported inferiorly by the splenocolic and splenophrenic ligaments [19].

The etiology of this condition may be congenital or acquired. The pathophysiologic mechanism of congenital WS in most medical literatures is due to failure of complete fusion of the mesogastrium and posterior abdominal wall during embryonic life, which may result absence or improper development of one or more ligament, and splenic hypermobility [8,9]. Acquired WS occur secondarily to trauma, multiparity, connective tissue diseases, renal agenesis, and hormonal changes after pregnancy, which lead to laxity or damage of suspensory ligaments [10].

The most frequent causes of WS in children are improper fixation and congenital maldevelopment [11]. A number of diseases, including renal agenesis, immunoglobulin deficiency, infectious mononucleosis, malaria, Gaucher disease, Hodgkin lymphoma, and DiGeorge syndrome, have been associated with acquired WS in pediatric patients [12].

Whether congenital or acquired, wandering spleen with torsion is a rare clinical entity, overall incidence is <0.2 % and it has a 2-peak incidence that mainly occurs in children aged between 3 months and 10 years, which is 2.5 times more common in male than females. Among adults most cases are seen between 20 and 40 years with female predominance [13,14].

The clinical presentation of wandering spleen varies widely, ranging from asymptomatic cases to acute abdominal emergencies or the presence of a palpable abdominal or pelvic mass. Notably, up to 50 % of patients remain asymptomatic [15]. In pediatric patients, the severity of symptoms largely depends on the degree of vascular pedicle torsion. Mild torsion may cause chronic, intermittent abdominal pain due to splenic congestion, while moderate torsion often leads to episodic pain resulted from recurrent twisting and untwisting of the pedicle. Severe torsion, however, can cause acute abdominal pain and splenic infarction, requiring urgent intervention [16]. The spleen is normally stabilized by several peritoneal attachments, with the splenogastric ligament containing the short gastric vessels being one of the most critical for maintaining its anatomical position [21].

We have gathered similar cases, regardless of age, using the keywords. Table 1 presents these cases with varying data, showing no recognizable pattern or definitive conclusion [[17], [18], [19], [20], [21], [22], [23], [24], [25], [26], [27], [28], [29]].

**Table 1:** Presents multiple related cases, comparing various factors.

The case	Age	Sex	Duration	Brief clinical presentation and imaging findings	Management and intraoperative findings
Romero, 2003 [27]	9 years	F	24 h	Acute pain in the left upper quadrant, associated with nonbilious emesis, started after eating and persisted. She has a history of 2 times splenomegaly after infection (EBV titers were elevated including IgG & IgM). She developed high-grade fever and significant leukocytosis after admission. Abdominal ultrasound with Doppler flow studies showed a moderately enlarged spleen and absence of flow in the splenic vein and distal splenic artery. Abdominal CT revealed an 11-cm hypoechoic spleen; suggestive of splenic infarction secondary to splenic vein thrombosis	At laparotomy, the spleen was found to be engorged and en- cased in the greater omentum with signs of infarction. The splenic pedicle was torsed >360° and involved the splenic vessels as well as the distal pancreas. The splenic ligaments could not be located. Splenectomy and distal pancreotectomy were performed
Karmazyn, 2005 [28]	The range was 4.2–15.3 years (the mean age was 9.7 years)	4 F 3 M	N/A	Abdominal pain associated with abdominal distension and vomiting. Abdominal ultrasound showed low-lying spleen and enlarged stomach occupying the splenic fossa	At surgery, the spleen was found anterior to the gastric antrum, causing gastric outlet obstruction. Splenopexy was performed
				Abdominal pain. Abdominal ultrasound showed low-lying spleen	Splenopexy was performed
				Abdominal pain. Abdominal ultrasound showed low-lying enlarged spleen	Splenopexy was performed
				Abdominal pain, in which the patient was additionally diagnosed later with acute pancreatitis. Abdominal ultrasound showed initially mild splenomegaly and a moderate amount of peritoneal pelvic fluid, then after a period, it showed low-lying spleen, confirming the diagnosis	Splenopexy was performed
				Recurrent abdominal pain. Abdominal ultrasound showed abnormal position of the spleen towards the mid-abdomen with enlargement and no flow	Torsion of wandering spleen was diagnosed, which was confirmed by surgery. Splenectomy was done subsequently
				Recurrent abdominal pain. Abdominal ultrasound showed enlarged spleen in the normal position, CT scan done after days showed enlarged spleen in abnormal position and confirming the diagnosis	Splenectomy was done
				The patient had polysplenia. Abdominal ultrasound showed enlarged low-lying one of the spleen, establishing its diagnosis as wandering spleen. Repeated ultrasound showed heterogenous mass without flow. CT scan after confirmed its cystic nature	Splenectomy was done
Ayaz, 2012 [18]	4 years	M	More than 7 months	Acute presentation of sudden severe abdominal pain on top of chronic presentation of vague abdominal pain, along with vomiting, fatigue and failure to thrive. On ultrasound, ectopic spleen was shown in the left paramedian region in the supine position, size was 80 × 39 mm at the first presentation (mild enlargement), with progressive enlargement months after	Emergency splenectomy was performed. Acute torsion of pre-existing ectopic spleen was confirmed by surgery
Olasehinde, 2014 [29]	6 years	M	36 h	Right lumbar abdominal pain, associated with right lumbar tender mass on examination. Abdominal ultrasound showed a mass with echogenicity consistent with that of the spleen with no blood flow and an empty splenic bed, raised the suspicion of a torsion of a wandering spleen	The presence of wandering spleen with torsion of the vascular pedicle was confirmed at laparotomy. Splenectomy was performed
	42 years	F	3 days	The patient was a pregnant in the 26-week of gestation. She had severe colicky lower abdominal pain. She had a background history of progressively increasing lower abdominal mass which was tender, misdiagnosed initially as a torsed ovarian cyst. Abdominal & pelvic ultrasound scan showed a heterogeneously hypoechoic mass overlying the uterus and the left ovary with no blood flow	The presence of wandering spleen with torsion of the vascular pedicle was confirmed at laparotomy. Splenectomy was performed
Chauhan, 2016 [26]	32 years	F	The last 2/3 days (with past history)	Intense left-sided abdominal pain associated with low-grade fever. A vague mass was palpated in the left mid-lower abdomen. Ultrasound & CT scan revealed absence of spleen in its normal location and a heterogeneous hypoechoic capsulated mass with size and shape resembling that of the spleen in the left mid and lower abdomen. Color Doppler imaging showed absence of internal vascularity	On surgery, the spleen appeared congested and infracted, and total splenectomy was performed
Anand, 2018 [20]	5 years	M	Brief duration of short time	Sudden onset of intense dull abdominal pain. Spleen is absent in its normal position, hypoechoic mass lesion in the left lower abdomen by the ultrasound with no internal vascularity by the Doppler study. CT scan confirmed the presence of an enlarged, minimally enhancing and inferiorly displaced spleen with characteristic ‘whirled appearance’ of the splenic hilum	Intra-operatively, the spleen was torsed 720° on its pedicle with an engorged and thrombosed splenic vein, the ligamentous attachments (gastro-splenic, splenocolic, splenophrenic and splenorenal) of spleen were absent leading to an inferiorly displaced wandering spleen
Assaf, 2019 [23]	13 years	F	2 days	Generalized acute abdominal pain (worst in the hypogastric region) with fever, anorexia and vomiting, prior history of mild abdominal pain and abdominal mass. Ultrasound showed absence of spleen in its normal position. A Doppler ultrasonography showed that the spleen was in the hypogastric region with a diameter of 14 cm and a very low perfusion	An emergent laparotomy showed the spleen was infarcted without any ligamentous attachments. The hilar vessels of the spleen were within a long mesentery
Wang, 2020 [19]	3 years	M	2 days	Abdominal pain along with vomiting, initially intermittent, then continuous and severe. CT scan with 3-dimensional reconstruction showed an enlarged displaced spleen occupying the left abdomen cavity, the distal end reaching the iliac crest. It also revealed a flexed elongated splenic vascular pedicle, which is shown as the whirl sign, with hyperdense parenchyma in the axial plane, suggesting the occurrence of splenic torsion	Emergent splenectomy was done, during the exploratory surgery, the absence of splenic suspensory ligaments was confirmed, and a 720°-twist vascular pedicle involving the tail of the pancreas in the torsion was identified
Koliakos, 2020 [21]	25 year	F	Several days	Epigastric pain with multiple times of vomiting. Ultrasound identified a splenomegaly as well as a small quantity of peritoneal effusion. CT scan confirmed the presence of a splenomegaly and demonstrated hypoperfusion of the enlarged spleen due to a volvulus of its vascular pedicle	During surgery, a torsion of the splenic vascular pedicle was discovered, secondary to the absence of the splenocolic and splenophrenic ligaments
Liao, 2022 [17]	6 years	F	1 week with worsening in the last 2 days	1 week history of spasmodically periumbilical pain worsened in the last two days, no fever, vomiting, diarrhea, constipation etc. Imaging showed homogeneous hyperechoic mass in the pelvis with absence of spleen in the normal site	Emergent laparoscopy then laparotomy was done and detorsion with replacement it into normal site and 2 stiches were performed. A torsed wandering spleen in 720° clockwise was identified
Tang, 2022 [25]	11 years	F	1 year with worsening in the last week	A year history of intermittent left lower quadrant abdominal pain, which had worsened in the last week, increased by movement, radiating to the back, lasting for many hours, associated with low-grade fever, abdominal distension and 3-day history of constipation. Abdominal radiograph demonstrated loss of the normal splenic shadow and left upper quadrant bowel dilatation. In addition to an ovoid opacity seen at the left iliac fossa suggestive of an abnormally located spleen. Ultrasound abdomen demonstrated the absence of spleen in the left hypochondrium and confirmed the presence of an enlarged spleen in the left iliac fossa. The spleen appeared diffusely hypoechoic with no internal Doppler signal demonstrated. Whirlpool appearance at the splenic hilum was present with absence of Doppler signal indicating torsion. Minimal intraabdominal free fluid was present.	An emergency laparotomy was arranged. Intra-operatively, the spleen was enlarged and it was occupying the left iliac fossa region. The spleen appeared non-viable, with tight torsion of the splenic hilum (twisted four times). It was untwisted and after a period of warming, there were no signs of recovery. Additionally, there was evidence of thrombosis of both splenic vein and artery; both were transfixed and divided and the spleen was subsequently removed.
Lugo-Fagundo, 2022 [22]	16 years	F	N/A	Severe periumbilical abdominal pain. CT scan revealed that the spleen was located in an inverted position in the right lower quadrant, with swirling of the mesenteric vessels leading to the splenic pedicle, as well as the splenic artery towards the splenic hilum in the right lower quadrant before a sudden cut-off	During surgery, the spleen was found to be torsed upon itself 720 degrees around an abnormal vascular pedicle
Ferrara, 2024 [24]	13 years	M	7 days	Hypogastric abdominal pain along with vomiting and tumefaction. homogeneous mass with no evidence of vascular flow on the color Doppler study and a maximum diameter of 20 cm and a clear plane of cleavage. From the cranio-caudal scan, we observed the characteristic swirling appearance (Whirlpool sign) of the arterial and venous vessels at the hilum (video) with marked regional venous dilatation	During surgery, a necrotic spleen was removed after ligation of the hilar vessels by laparoscopic technique

F: Female, M: Male, N/A: Not available, EBV: Epstein-Barr virus.

Torsion with prolonged venous occlusion can cause perisplenitis, localized peritonitis, adhesions, venous thrombosis, and hypersplenism. Torsion with arterial occlusion may result in hemorrhagic infarction, subcapsular and intrasplenic hemorrhage, gangrene, degenerative cysts, and functional asplenism [18].

In our case, the presentation was also unusual, mimicking perforated acute appendicitis, with diffuse abdominal pain, positive peritoneal signs, and severe tenderness in the right lower quadrant (RLQ). However, ultrasound effectively ruled out appendicitis and gynecologic differential diagnoses, emphasizing the crucial role of ultrasound imaging in evaluating emergent abdominal conditions.

The presentation was acute on top of chronic. This can be explained by the long-standing recurrent abdominal pain caused by moderate splenic torsion, resulting in splenic congestion, or the recurrent torsion-detorsion process. This was followed by a severe torsion that could not be spontaneously detorsed. The anemia may be attributed to acute splenic congestion from the torsion, though it was not low enough to suggest splenic rupture. Additionally, blood pressure remained nearly normal, which could also exclude the splenic rupture risk. The transient fever, mild leukocytosis, and elevated CRP likely resulted from the acute inflammatory process associated with the torsion. Tachycardia could be a consequence of either the severe pain or the fever.

While wandering spleen is considered uncommon in pediatric females, several recent case reports have described its occurrence in this population; so we have collected many relevant recent cases including this group entity of cases as shown in Table 1.

## 4 Conclusion

Wandering spleen is a rare condition with various etiologies, often resulting in splenic torsion and acute abdominal emergencies. Early diagnosis is achieved by abdominal ultrasound showing an ectopic spleen and the “whirlpool sign” is crucial to prevent complications such as infarction and rupture. Ultrasound and contrast-enhanced CT are used in complex cases. Management depends on splenic viability; splenectomy is indicated in cases of significant infarction, while splenopexy may be considered if tissue is viable. Timely intervention is essential to reduce morbidity and mortality.

## List of abbreviations

WS: wandering spleen
RLQ: right lower quadrant
CT: computed tomography
CRP: c-reactive protein
PD: postoperative day
ED: emergency department
OR: operating room
MRI: magnetic resonance imaging
NPO: nil per os (nothing by mouth)
POD: postoperative day
Hct: hematocrit

## Author contribution

Yara al Najajreh, Alaa R. AL-Ihribat, Kareem Ibraheem, Ayat Aljuba: Conceptualization, case analysis, manuscript writing, and editing.

Bahaa AbuRahmeh, Alaa R. AL-Ihribat, Noor Khashan: Data collection, literature review, and manuscript drafting.

Yara al Najajreh, Ayat Aljuba, Bahaa AbuRahmeh: Clinical management of the patient, data interpretation, and manuscript revision. All authors have read and approved the final manuscript and agree to be accountable for all aspects of the work.

## Consent

Written informed consent was obtained from the patient parents for the publication of this case report and its accompanying images. A copy of the consent form is available for review by the Editor-in-Chief upon request.

## Ethical approval

Ethical approval was not applicable for this study, as our institution's IRB committee at Palestine Polytechnic University does not mandate approval for reporting individual cases or case series

## Guarantor

Yara al Najajreh is the guarantor for this study, taking full responsibility for the research and its outcomes. She had access to all the data and made the final decision to publish the study.

## Research registration number

None.

## Funding

This research did not receive any specific grants from funding agencies in the public, commercial, or not-for-profit sectors.

## Conflict of interest statement

The authors have no conflict of interest to declare.

## Data Availability

The data used to support the findings of this study are included in the article.
